# Resistance to SARS-CoV-2 infection in camelid nasal organoids is associated with lack of ACE2 expression

**DOI:** 10.1038/s44298-024-00054-0

**Published:** 2024-09-02

**Authors:** Tim I. Breugem, Samra Riesebosch, Debby Schipper, Anna Z. Mykytyn, Petra van den Doel, Joaquim Segalés, Mart M. Lamers, Bart L. Haagmans

**Affiliations:** 1 Viroscience Department, Erasmus Medical Center, Rotterdam, The Netherlands; 2 Unitat Mixta d’Investigació IRTA-UAB en Sanitat Animal, Centre de Recerca en Sanitat Animal (CReSA), Campus de la Universitat Autònoma de Barcelona (UAB), 08193 Bellaterra, Barcelona Spain; 3 Departament de Sanitat i Anatomia Animals, Facultat de Veterinària, UAB, 08193 Bellaterra, Barcelona Spain; 4 Present Address: Infection and Immunity, Amsterdam University Medical Center, Amsterdam, The Netherlands; 5 Present Address: Programme of Emerging Infectious Diseases, Duke-NUS Medical School, Singapore, Singapore

**Keywords:** Restriction factors, SARS-CoV-2, Viral reservoirs, Virus-host interactions

## Abstract

The severe acute respiratory syndrome coronavirus 2 (SARS-CoV-2) infects a variety of animal species. Susceptibility to SARS-CoV-2 is primarily determined by the utilization of the viral receptor, ACE2. SARS-CoV-2 can utilize a broad range of animal ACE2 isoforms in vitro, including the ACE2 from various camelid species. However, experimental infection of these animals does not lead to productive infection or seroconversion. In this study, we investigate the susceptibility of camelids to SARS-CoV-2 using novel well-differentiated camelid nasal organoids. We show that camelid nasal organoids are highly susceptible to Middle East respiratory syndrome coronavirus (MERS-CoV) infection, but not to infection with different SARS-CoV-2 variants (614G, BA.1 or EG.5.1.1). All viruses efficiently infected human airway organoids. Immunohistochemistry analysis revealed the absence of ACE2 on camelid nasal organoids and dromedary camel upper respiratory tract. In contrast, DPP4 was expressed in both camelid nasal organoids and the camel upper respiratory tract, which correlates with MERS-CoV infection. This study indicates that the camelid upper respiratory tract lacks expression of ACE2, which is associated with resistance to SARS-CoV-2 infection.

## Introduction

Coronaviruses are present across a diverse range of animal species and occasionally spillover into the human population^1,2^. These cross-species transmissions are rare but have resulted in the emergence of multiple pathogenic coronaviruses over the past two decades, including Middle East respiratory syndrome coronavirus (MERS-CoV) in Saudi Arabia in 2012 and severe acute respiratory syndrome coronavirus 2 (SARS-CoV-2) in China late 2019^3–5^. After its emergence, SARS-CoV-2 swiftly spread globally causing the COVID-19 pandemic. The ongoing circulation and evolution of SARS-CoV-2 has led to the emergence of a huge number of variants. Viruses from the Omicron lineage show substantial genetic and phenotypic differences from early SARS-CoV-2 isolates^6–11^. To date, these viruses remain the predominant SARS-CoV-2 strains globally, continually giving rise to novel variants^12^.

SARS-CoV-2 likely originated from bats and exhibits a broad host range, with documented natural and experimental infections in various non-human primate species, cervids, mustelids, felids, and muroids^13–15^. The spillback of SARS-CoV-2 into animals poses a potential health hazard due to reservoir formation. This is illustrated by the SARS-CoV-2 circulation in deer and mink^16–20^. Camels are of significant interest as potential reservoirs for SARS-CoV-2, particularly due to the prevalence of MERS-CoV in these animals and the potential of recombination with SARS-CoV-2^21–23^. MERS-CoV infects camel cells via the dipeptidyl peptidase 4 (DPP4) receptor which is readily available in the camel upper respiratory tract, facilitating MERS-CoV replication and spread in camel populations^24,25^.

SARS-CoV-2 uses the angiotensin converting enzyme 2 (ACE2) receptor to enter cells and therefore animal susceptibility is largely determined by ability of this virus to utilize of animal ACE2^26–29^. SARS-CoV-2 has been shown to use a plethora of animal ACE2 receptors^30^, including the ACE2 from various camelid species such as dromedary camels, Bactrian camels and alpacas^27,31^. However, in vivo infection with SARS-CoV-2 has not shown productive replication in alpacas^32^. Furthermore, primary airway cultures from Bactrian camels and llamas were not susceptible to SARS-CoV-2 infection^33^. Such results suggest that in the camelid airways there are other barriers to infection beyond ACE2 utilization. Interestingly, recent serological evidence from dromedary camels in Oman suggests that Omicron strains naturally infect camelids^34^. This finding suggests a host range expansion of the emerging SARS-CoV-2 Omicron variants for camelids, although confirmative studies are still lacking. Host range expansion has been observed for SARS-CoV-2 pre-omicron variants, that acquired mutations which induced replication in muroids compared to the wild-type virus^35^.

In this study, we aimed to study whether emerging SARS-CoV-2 Omicron variants could infect camelids using relevant in vitro models. To this end, we successfully established and well-differentiated camelid nasal organoids to accurately model the camelid upper respiratory tract epithelium. The nasal epithelium is regarded as the primary replication site for SARS-CoV-2 and is thought to be important for its dissemination to other parts of the respiratory tract and lung^36^. We utilized these organoids to characterize the susceptibility of the camelid upper respiratory tract to the emerging SARS-CoV-2 Omicron variants and MERS-CoV, and to identify potential barriers to infection.

## Results

### MERS-CoV targets multiciliated cells and causes deciliation in camelid nasal organoids

**Fig. 1:**
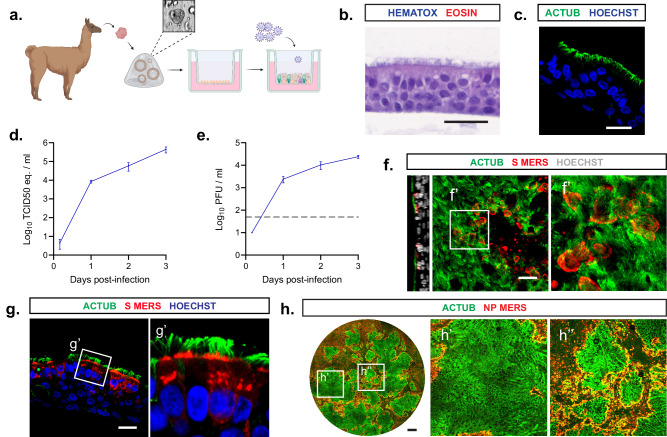
MERS-CoV targets multiciliated cells and causes deciliation in camelid nasal organoids. **a**, **b** Establishment of camelid nasal organoids (**a**, created with BioRender.com) and cells were stained (**b**) with hematoxylin and eosin. **c** Camelid nasal organoids were stained for cilia (ACTUB, green) and nuclei (Hoechst, blue). (**d**, **e**) Camelid nasal organoids were infected with MERS-CoV with a moi of 0.1 and viral replication was determined by **d** RT-qPCR and **e** plaque assay titration. Data is depicted as mean with SEM. Dotted line shows detection limit. **f**, **g** Infected cells on transwell inserts (**f**) and paraffin-embedded inserts (**g**) were stained at 1 dpi for cilia (ACTUB, green) and MERS-CoV spike (S, red). **h** Full well imaging was performed on MERS-CoV infected camelid nasal organoid inserts at 3 dpi for cilia (ACTUB, green) and MERS-CoV nucleoprotein (NP, red) to show deciliation. Experiments were repeated at least once. Scale bars are 20 µm (**b**, **c**, **f**, **g**) and 500 µm (**h**). Inserts (**f**’, **g**’, **h**’ and **h**”) show digital zoom of the original image.

To model coronavirus infections in the camelid upper respiratory tract, we have successfully isolated self-renewing nasal organoids from alpaca nasal tissues using an adapted protocol from Sachs et al.^37^, as described previously^37–39^. In short, camelid basal cells were isolated from the nasal turbinates of healthy alpacas and maintained in basement membrane matrix droplets to form spherical three-dimensional organoids (Fig. 1a). Next, the cells were plated on transwell inserts and differentiated on air–liquid interface (ALI) for 10–14 days until they formed a well-differentiated pseudostratified respiratory epithelium on the flat surface of the transwell (Fig. 1b) with abundant ciliated cells (Fig. 1c). All subsequent experiments were performed on ALI-differentiated camelid nasal organoids grown on transwells. To investigate whether camelid nasal organoids efficiently model coronavirus infection, they were infected with MERS-CoV at a moi of 0.1 and viral replication was assessed by RT-qPCR and plaque assay titration (Fig. 1d, e). MERS-CoV efficiently replicated in these cells and reached peak titres of ~10^4^ PFU per mL at 3 days post infection (dpi) (Fig. 1d, e). Next, we performed confocal microscopy at 1 dpi and showed that MERS-CoV targeted mainly multiciliated cells (Fig. 1f, g). Furthermore, we observed severe loss of ciliated cells in the camelid nasal organoids at 3 dpi (Fig. 1h), which is similarly observed in vivo^40^. These data demonstrate that our camelid nasal organoids faithfully model MERS-CoV infection and that they are a reliable tool to characterize coronavirus infections in camelids.

### SARS-CoV-2 variants 614G, BA.1 and EG.5.1.1 cannot replicate in camelid nasal organoids

**Fig. 2:**
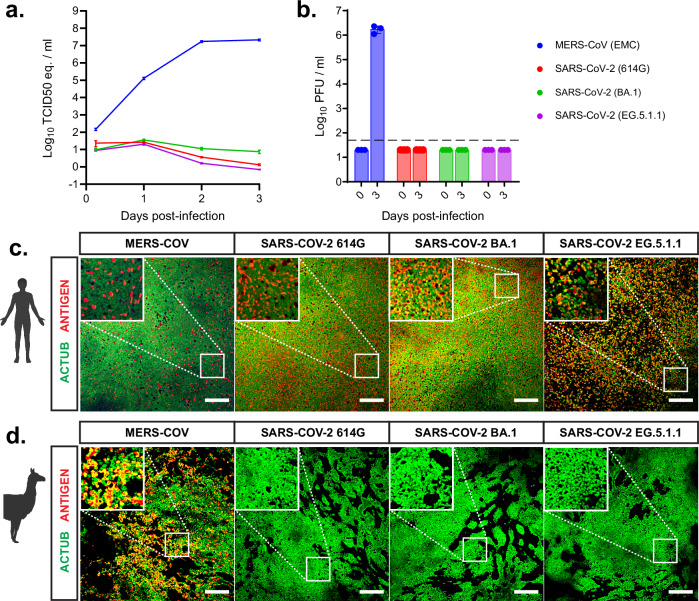
SARS-CoV-2 variants 614G, BA.1 and EG.5.1.1 cannot replicate in camelid nasal organoids. Camelid nasal organoids and human airway organoids were infected with MERS-CoV and SARS-CoV-2 614G, BA.1 and EG.5.1.1 variants with a moi of 0.1 to assess camelid susceptibility. **a**, **b** Viral replication of SARS-CoV-2 variants 614G, BA.1 and EG.5.1.1 in camelid nasal organoids was determined by **a** RT-qPCR and **b** plaque assay titration. Data is depicted as mean with SEM, and for (**b**) values of the individual replicates were shown. Dotted line shows detection limit. **c**, **d** Infected inserts of **c** human airway organoids and **d** camelid nasal organoids were stained at 3 dpi for ciliated cells (ACTUB, green) and viral antigen (Spike for MERS-CoV and Nucleoprotein for SARS-CoV-2, red). Illustrations were created with BioRender.com. Experiments were repeated at least once. Scale bars are 200 µm. Inserts (squares) show digital zoom of the original image.

To investigate the tropism of novel Omicron-lineage SARS-CoV-2 isolates for the camelid respiratory epithelium, we infected the camelid nasal organoids with the SARS-CoV-2 variant 614G from early 2020, SARS-CoV-2 Omicron variant BA.1 from late 2021 and EG.5.1.1 from 2023 with a moi of 0.1 (Fig. 2). MERS-CoV was used as positive control for camelid replication throughout the experiments. We show that SARS-CoV-2 614G could not replicate in the camelid nasal organoids (Fig. 2a, b). BA.1 and EG.5.1.1. were similarly unable to productively infect these cells (Fig. 2a, b). Next, we performed confocal microscopy at 3 dpi in human and camelid organoids to investigate whether SARS-CoV-2 could infect camelid cells (Fig. 2c, d). Whereas both MERS-CoV and all SARS-CoV-2 variants showed efficient infection in human airway organoids (Fig. 2c), we did not detect any SARS-CoV-2-infected cells in the camelid nasal organoids (Fig. 2d). These data demonstrate that neither the SARS-CoV-2 614G nor the Omicron-lineage SARS-CoV-2 isolates can infect the camelid upper respiratory tract, prompting us to investigate receptor expression.

### DPP4 but not ACE2 is expressed in camelid nasal organoids and tissue

**Fig. 3:**
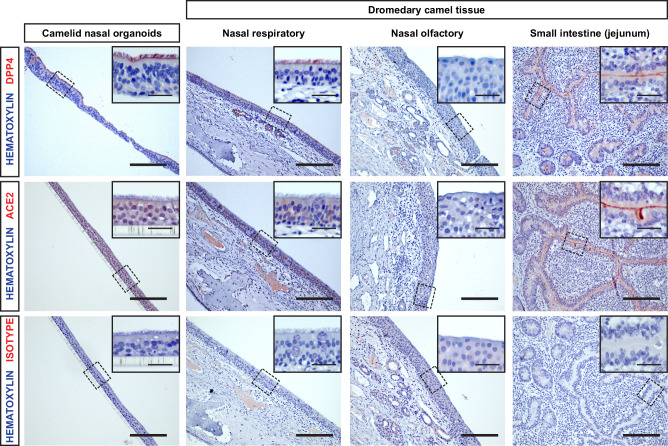
DPP4 but not ACE2 is expressed in camelid nasal organoids and tissue. Immunohistochemistry for DPP4 (top), ACE2 (middle) and isotype (bottom) was performed on camelid nasal organoids and dromedary camel nasal and intestinal tissues from four donors. Representative images at ×20 magnification were shown of one donor. Scale bars are 100 µm. Inserts (squares) show digital zoom of images of the same region at ×40 magnification, scale bars are 20 µm.

**Fig. 4:**
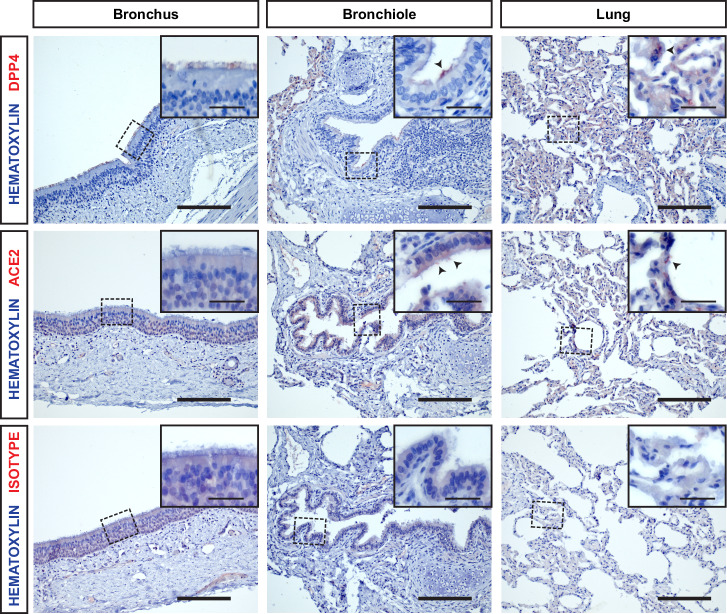
ACE2 is expressed sporadically in the dromedary camel lower respiratory tract. Immunohistochemistry for DPP4 (top), ACE2 (middle) and isotype (bottom) was performed on dromedary camel bronchus, bronchiole and lung tissue of four donors. Representative images at ×20 magnification were shown of one donor. Scale bars are 100 µm. Inserts (squares) show digital zoom of images of the same region at ×40 magnification, scale bars are 20 µm.

Receptor expression and utilization (i.e. DPP4 for MERS-CoV and ACE2 for SARS-CoV-2) are major determinants for the susceptibility to coronavirus infection. As it has been shown previously that the ACE2 of camelid species can be utilized by SARS-CoV-2 for viral entry^27,31^, we hypothesized that the block in SARS-CoV-2 susceptibility was associated with the ACE2 expression pattern in the camelid respiratory epithelium. To investigate this, we performed immunohistochemistry for DPP4 and ACE2 on the camelid nasal organoids and dromedary camel tissues of the nasal respiratory epithelium, nasal olfactory epithelium, bronchus, bronchiole, lung and small intestine (jejunum) from four donors (Figs. 3 and 4). Dromedary camel olfactory epithelium was available in three out of four donors. DPP4 was efficiently expressed in the cilia of the camelid nasal organoids, which correlates to MERS-CoV replication (Fig. 3). In the dromedary camel nasal tissues, DPP4 was expressed on ciliated cells and glandular cells of the respiratory epithelium, but not on the olfactory epithelium (Fig. 3). Furthermore, DPP4 was expressed in the small intestine and bronchus, and on some bronchiolar and lung cells (Figs. 3 and 4). In contrast, ACE2 was not expressed in the camelid nasal organoids and throughout the dromedary camel upper respiratory tract (Fig. 3). Although the ACE2 staining shows a faint signal in the cytoplasm and the nucleus of some cells, ACE2 was completely absent on the surface of the upper respiratory tract epithelium. ACE2 was efficiently expressed in the small intestine of dromedary camels (Fig. 3). This shows that the ACE2 antibody efficiently detected the camelid ACE2 isoform. Furthermore, ACE2-positive cells were observed in the dromedary camel lung in three out of four donors, and some cells that faintly expressed ACE2 in the bronchiole of all donors (Fig. 4). The isotype control showed limited background staining (Figs. 3 and 4). These data indicate that the lack of ACE2 expression in the camelid upper respiratory tract is likely the main barrier preventing camelid infections with SARS-CoV-2.

## Discussion

In this study, we have established self-renewing camelid nasal organoids that model the in vivo tissue faithfully. Using these cells, we show that the lack of camelid susceptibility for SARS-CoV-2 infection is associated with the absence of ACE2 expression in the upper respiratory tract. We show that MERS-CoV infection in the camelid nasal organoids is efficient and correlates with DPP4 expression. MERS-CoV infects mainly multiciliated cells and causes severe loss of ciliated cells in the camelid nasal organoids. These observations are in accordance with earlier in vitro and in vivo MERS-CoV tropism studies in camelids^25,40–42^. For SARS-CoV-2, deciliation might be an effect of dedifferentiation of multiciliated cells and has been linked with virus dissemination to lower parts of the lung due to decreased mucociliary clearance^43^. Whether similar cellular mechanisms induce deciliation of MERS-CoV-infected cells and the effect on MERS-CoV replication and pathogenesis remains to be determined. Taken together, the tissue histology, the expression pattern of the coronavirus receptors and the accurate modelling of MERS-CoV infection show that the camelid nasal organoids model the camelid upper respiratory tract well and provide a reliable model for coronavirus tropism studies.

The camelid upper airway is not susceptible to SARS-CoV-2 infection. Previous in vitro and in vivo infection studies have shown that the camelid airway could not be infected with early SARS-CoV-2 variants from 2020^32,33^. However, with the emergence of the genetically distant Omicron viruses late 2021, the susceptibility of camelids to SARS-CoV-2 needed to be investigated further. One study suggests serological evidence for SARS-CoV-2 infection in dromedary camels from Oman in 2021^34^, however without controlling for MERS-CoV cross-neutralization it remains difficult to interpret these results. Previous work has shown that the camel ACE2 can be bound and utilized for infection by the SARS-CoV-2 spike protein, using cell lines, overexpression assays and pseudoviruses^27,31,44^. We show that both SARS-CoV-2 614 G and the Omicron strains BA.1 and EG.5.1.1 cannot replicate in camelid nasal organoids, indicating that the Omicron viruses did likely not change their tropism for the camelid upper respiratory tract. These data highlight that ACE2-binding or utilization assays do not always correlate with productive SARS-CoV-2 infection of relevant cell types, due to the potential lack of ACE2 expression on the apical membrane. This should be taken into account when conducting species susceptibility screening based on receptor utilization assays.

The lack of susceptibility of camelids to SARS-CoV-2 experimental infection correlates with the lack of ACE2 expression in camelid upper respiratory tract tissues. In humans, the expression of ACE2 in the upper respiratory tract is considered a hallmark of its widespread community transmission and pandemic nature. Similarly, in the SARS-CoV-2 susceptible animals—such as hamsters and mink—the ACE2 expression pattern closely resembles that of humans^45,46^. In this study, we show that ACE2 is not expressed on the apical surface of the camelid nasal organoids and camel nasal tissues. We show that ACE2 is detected to low levels in the cytoplasm and in the nucleus of these cells. Nuclear ACE2 has also been detected in human cells^47,48^. One other study has investigated the expression of ACE2 in the respiratory tract of camelid species, including Bactrian camels, alpacas and llamas^46^. These authors show that most camelid respiratory tissues did not express ACE2, except for expression on Bactrian camel pneumocytes^46^, similar to our results. In contrast to our data, they demonstrate sparse expression of ACE2 on alpaca nasal cells^46^. Although the expression of ACE2 in the camelid lower respiratory tract is very limited, future studies are needed to determine the tropism of SARS-CoV-2 for this anatomical area. The differential expression of ACE2 in mammal respiratory tissues is a major determinant for SARS-CoV-2 susceptibility, but regulatory mechanisms behind this still remain largely unknown and warrant further investigation.

ACE2 is expressed in the intestinal tract of camelids. Although gastroenteric replication of SARS-CoV-2 is well established in humans and correlates with high ACE2 expression in the human intestine^38,49–51^, ACE2 expression in the intestinal tract of animal species seems a poor determinant for productive SARS-CoV-2 replication^45,46,52^. ACE2 is abundantly expressed in the intestinal tract of non-susceptible species^45,46^, such as camelids, indicating that the intestines are likely not a primary replication site of SARS-CoV-2 in these animals and that the fecal-oral transmission route is inefficient. Interestingly, both SARS-CoV-2 and MERS-CoV likely originate from bat coronaviruses with a primary enteric tropism^26,53,54^, but seemed to have lost the ability to efficiently replicate in the intestinal tract of some species when they cross the species barrier, even in the presence of its receptor.

In conclusion, our study shows that camelids provide an unlikely reservoir for coronaviruses that utilize the ACE2 receptor such as SARS-CoV-2, while these animals are more permissive to coronaviruses that utilize DPP4 such as MERS-CoV. Future studies should focus on unravelling the determinants of ACE2 expression in different animal species, as this might provide novel insights into the zoonotic emergence and transmission of pathogenic coronaviruses such as SARS-CoV-2 and other ACE2-utilizing coronaviruses.

## Materials and methods

### Cell culture

Vero (WHO, RCB 10-87) and VeroE6 cells (ATCC, CRL 1586TM) were maintained in DMEM supplemented with 10% foetal calf serum (FCS), HEPES (Invitrogen), sodium bicarbonate (0.075%, Gibco), penicillin (100 IU/mL), and streptomycin (100 IU/mL) at 37 °C with 5% CO_2_. Calu-3 cells (ATCC, HTB 55) were maintained in OptiMEM (1X; Invitrogen) supplemented with 10% FCS, penicillin (100 IU/mL), and streptomycin (100 IU/mL) at 37 °C in a humidified CO_2_ incubator. Cells were passaged when 70–80% confluent and routinely checked for mycoplasma. When Calu-3 cells were plated complete medium was supplemented with y-27632 (1 µM; MedChemExpress) and refreshed after 48 h for complete medium without y-27632. Cells were used to produce virus stocks and virus titration.

### Viruses

SARS-CoV-2 (isolate BetaCoV/Munich/BavPat1/2020; OM304632; European Virus Archive Global #026 V-03883; EVAg) was isolated on VeroE6 cells in OptiMEM supplemented with GlutaMAX, penicillin (100 IU/mL), and streptomycin (100 IU/mL) and the virus was subsequently passaged up to passage 3 on Calu-3 cells in ADMEM supplemented with GlutaMAX, penicillin (100 IU/mL), and streptomycin (100 IU/mL) at 37 °C in a humidified CO_2_ incubator. The SARS-CoV-2 BA.1 (isolate SARS-CoV-2/human/NLD/EMC-Omicron-1/2021; OM287553) was propagated and passaged up to passage 3 on Calu-3 cells in ADMEM supplemented with GlutaMAX, penicillin (100 IU/mL), and streptomycin (100 IU/mL). The SARS-CoV-2 EG.5.1.1. (isolate hCoV-19/France/GES-IPP15954/2023, lineage EG.5.1.1 kindly provided by Dr. Etienne Simon-Loriere) was propagated on VeroE6-TMPRSS2 cells up to passage 2 and to passage 3 on Calu-3 cells in ADMEM supplemented with GlutaMAX, penicillin (100 IU/mL), and streptomycin (100 IU/mL). MERS-CoV (isolate EMC, genbank accession no. NC019843) was propagated on Vero cells in OptiMEM (1x) + GlutaMAX, penicillin (100 IU/mL), and streptomycin (100 IU/mL). Stocks were produced as described previously^38,55^, and titrated by plaque assay titration or TCID50 method. All viruses were sequence verified by illumina deep sequencing and did not exhibit cell culture adaptations. All work with infectious SARS-CoV-2 and MERS-CoV was performed in a Class II Biosafety Cabinet under BSL-3 conditions at Erasmus Medical Center

### Isolation, culture and differentiation of camelid nasal organoids

Camelid nasal organoids were isolated from the nasal turbinates of 6–8-month-old alpacas (Vicugna pacos) that were purchased by private sale and were utilized as healthy control animals in a previously published study^56^. Samples were obtained from animals that were euthanized with an overdose of Pentobarbital (50 mg/kg, for a total volume of 3–4 ml of a solution of 400 mg/ml) followed by exsanguination. Animal experiments were approved by the Ethical and Animal Welfare Committee of IRTA (CEEA-IRTA) and by the Ethical Commission of Animal Experimentation of the Autonomous Government of Catalonia (file N˚ FUE-2018-00884575 –Project N˚10370). Nasal turbinates were taken from the middle location, approximately 5 cm deep from the nostrils. Isolation of the nasal stem cells was performed using a protocol adapted from Sachs et al.^37^, as described previously^37–39^. In short, the nasal turbinates were cut into 2–4 mm pieces, washed in Advanced Dulbecco’s modified Eagle’s medium (ADMEM) supplemented with GlutaMAX (Gibco), HEPES (Invitrogen) and primocin (50 µg/mL; Invitrogen) (ADMEM+++), and incubated with Dispase (Corning) supplemented with y-27632 (5 µM; MedChemExpress) for 1 h at 37 °C to dissociate the epithelium. The digested tissue was mechanically sheared sequentially by pipetting up and down with a 5 mL pipet and P1000 pipet and filtered using a 100 µM cell strainer (Corning). The sheared and filtered tissue was washed three times with ADMEM+++ and resuspended in 300 µL of Basement Membrane Extract type-2 Reduced Growth Factor Select (BME-2; Biotechne) and plated in ~30 µL droplets in a 48-well suspension culture plate (Greiner). After solidification of the BME-2, 300 µL airway organoid (AO) medium was added to each well and the cells were cultured at 37 °C with 5% CO_2_ to form 3D camelid nasal organoids.

To obtain well-differentiated camelid nasal organoid the 3D camelid nasal organoids were dissociated into single cells using TrypLE Express (Invitrogen) and seeded on 12 or 24 well CELLTREAT® 0.4 µm Polyethylene Membrane Inserts (STEMCELL) coated with Rat Tail collagen type-I (Thermo Fisher) in AO and complete base medium (CBM; STEMCELL Pneumacult-ALI) in a 1:1 ratio at 37 °C with 5% CO_2_ to form a 2D monolayer. When confluent the 2D monolayer was cultured at air–liquid interface and the medium was refreshed for CBM. Medium was replaced every 5–7 days. The monolayers were differentiated at ALI for 10–14 days until a well-differentiated pseudostratified respiratory epithelium formed and the camelid nasal organoids were used for subsequent experiments.

### Isolation, culture and differentiation of human airway organoids

Adult lung tissue was obtained from residual, tumour-free material obtained at lung resection surgery for lung cancer. The Medical Ethical Committee of the Erasmus MC Rotterdam granted permission for this study (METC 2012–512). Informed consent was waived by the Medical Ethical Committee as all donor materials were completely anonymized and non-identifiable. Bronchiolar tissue was carefully dissected from distal lung parenchyma material to generate human airway organoids. The isolation, culture, and differentiation of these cells was performed according to a protocol as described previously^38,39^. The cells were differentiated at ALI for at least 6 weeks.

### Virus infection of camelid nasal organoids and human airway organoids

The camelid nose organoids and human airway organoids were infected on ALI as described previously^38,39^. In short, ALI monolayers were washed three times before inoculation on the apical side with 200 µL ADMEM+++ to remove any mucous and were infected with an multiplicity of infection (moi) of 0.1 in 200 µL ADMEM+++ for 2 h 37 °C with 5% CO_2_. The moi was calculated from the PFU titres of the corresponding stocks. The infected cells were washed three times with 200 µL ADMEM+++ to remove inoculum before taking the 2 h timepoint. Next, timepoints were taken every 24 h by washing the apical side with 200 µL ADMEM+++ for 10 min. The supernatant was stored at −80 °C until thawed for RNA isolation and plaque assay titration. Cells were fixed at 24 or 72 h post infection (hpi) for immunostaining.

### Viral titre determination by qRT-PCR

Viral RNA was isolated from infectious virus samples and was according to previously published protocol^38^. In short, 60 μl of sample was lysed in 90 μl of MagNA Pure LC Lysis buffer (Roche). The lysed samples were incubated for 15 min with 50 μl of Agencourt AMPure XP beads (Beckman Coulter). The beads were washed twice with 70% ethanol on a DynaMag-96 magnet (Invitrogen) and eluted in 50 μl of diethylpyrocarbonate-treated water. Quantitative reverse transcription polymerase chain reaction (PCR) was performed using primers targeting the E gene and comparing the Ct values with a standard curve derived from a virus stock titrated (TCID50 method) on Vero cells (MERS-CoV) or VeroE6 cells (SARS-CoV-2).

### Live virus titration

MERS-CoV virus stocks were titrated on either Vero cells by TCID50 method or plaque assay titration on Calu-3 cells. SARS-CoV-2 stocks were titrated on either VeroE6 cells by TCID50 method or plaque assay titration on Calu-3 cells. SARS-CoV-2 and MERS-CoV infectious samples from organoid infection experiments were exclusively titrated by plaque assay titration on Calu-3 cells to minimize variability between viruses.

Virus titration by TCID50 was performed as described previously^38^. Briefly, samples were thawed and centrifuged at 2000 × *g* for 5 min. Next, 10-fold serial dilutions were prepared for each sample in 6 replicates in OptiMEM (1x) + GlutaMAX. Aliquots of each dilution were added to monolayers of 20,000 Vero cells (for MERS-CoV) or VeroE6 cells (for SARS-CoV-2) in the same medium in a 96-well plate. The infected cells were incubated at 37 °C with 5% CO_2_ for 4–6 days and then examined for cytopathic effect. The TCID50 was calculated according to the method of Spearman & Kärber.

Virus titration by plaque assay on Calu-3 cells was performed as described previously^55^. Briefly, samples were thawed and centrifuged at 2000 × *g* for 5 min. Next, 10-fold serial dilutions were prepared for each sample in 200 µL OptiMEM (1x) + GlutaMAX supplemented with penicillin (100 IU/mL), and streptomycin (100 IU/mL) in a 96-well plate. 100 µL of each dilution was added to a monolayer of Calu-3 cells in the same medium in a 24-well plate and incubated for 2–4 h at 37 °C with 5% CO_2_. Medium was refreshed for 1.2% Avicel (FMC BioPolymer) in Opti-MEM I (1×) + GlutaMAX (Gibco) and incubated for 48 h and were fixed in formalin, permeabilized in 70% ethanol, and washed in phosphate-buffered saline (PBS). Cells were stained by incubation with rabbit-anti-nucleoprotein SARS-CoV-2 (Sino Biological; 40143-T62; 1:5000) or rabbit-anti-spike MERS-CoV (Sino Biological; 40069-T52; 1:5000) in PBS containing 0.6% BSA (bovine serum albumin; Sigma-Aldrich), washed with PBS and incubated with goat-anti-rabbit Alexa Fluor 488 (Invitrogen; A32731; 1:5000) in PBS containing 0.6% BSA. Plates were then washed again in PBS and scanned on the Amersham Typhoon Biomolecular Imager (channel Cy2; resolution, 10 mm; GE Healthcare).

### Immunofluorescent staining and immunohistochemistry

Immunofluorescent staining and immunohistochemistry on organoid transwells or animal tissue was performed as described previously^38,57^. Transwell inserts were fixed in formalin, permeabilized in 70% ethanol, and blocked for 60 min in 3% BSA in PBS (blocking buffer). For immunofluorescent staining on embedded inserts, transwell inserts were formalin-fixed, paraffin-embedded, sectioned and deparaffinized prior to blocking as described before^58^. Next, the cells were incubated with the corresponding primary antibodies with rabbit-anti-nucleoprotein SARS-CoV-2 (Sino Biological; 40143-T62; 1:1000), rabbit-anti-spike MERS-CoV (Sino Biological; 40069-T52; 1:1000), rabbit-ant-nucleoprotein MERS-CoV (GeneTex; GTX134868; 1:1000) and/or conjugated mouse-anti-acetylated alpha tubulin-488 (ACTUB; Santa Cruz; sc-23950; 1:200) overnight at 4 °C in blocking buffer, washed three times with PBS and incubated with goat-anti-rabbit Alexa Fluor 594 (Life Technologies; A11012; 1:1000) in blocking buffer for 2 h at room temperature. Cells were washed two times with PBS, incubated with Hoechst, washed twice with PBS, and mounted in Prolong Antifade (Invitrogen) mounting medium. Samples were imaged on a LSM700 confocal microscope using ZEN software (Zeiss). Representative images were acquired and shown as Z-projections, single slices or XZ cross sections.

For immunohistochemistry, organoid transwell inserts or dromedary tissue was formalin-fixed, paraffin-embedded, sectioned and deparaffinized prior to staining as described before^58^. Tissue sections were obtained from 6–8-month-old dromedary camels from a previously published study^59^. Samples were obtained from animals that were sedated with Midazolam (5 mg/ml) intramuscularly, with a dose depending on the weight of the animals (between 4 and 6 ml per animal), followed by euthanasia with an overdose of Pentobarbital (50 mg/kg, for a total volume of 20 ml of a solution of 400 mg/ml) intravenously followed by exsanguination. Animal ethics approval for the experiment was obtained, with the reference number 8003-2014 (Generalitat de Catalunya, Spain). ACE2 and DPP4 were stained using goat-anti-hACE2 (R&D Systems; AF933; 1:100) or goat-anti-hDPP4 (R&D Systems; AF1180; 1:100) and visualized with rabbit-anti-goat (Dako; P0160; 1:200) horseradish peroxidase labelled secondary antibody. The AF933 antibody was raised against human ACE2 and shows reactivity to hamster, rat and mouse ACE2 with sequence similarities of ~80% to human ACE2, similar to that of camelids (Table 1). Nuclei were visualized by haematoxylin staining. Concentration-matched normal goat IgG isotype staining (R&D Systems; AB-108-C) was performed to control immunohistochemistry experiments. Samples were imaged on Axio Imager.A2 using Zen software (Zeiss) using the indicated magnification. White balance and contrast were adjusted accordingly in the Zen software.

**Table 1:** Amino acid sequence similarity between human and animal ACE2 isoforms

ACE2 isoform	Sequence similarity to human ACE2 (%)
Mouse	80.3
Rat	80.7
Hamster	83.1
Bactrian camel	81.6
Wild Bactrian camel	81.6
Alpaca	81.8
Mink	81.2

Sequence similarities of animal ACE2 isoforms to human ACE2 were calculated from an amino acid alignment and were presented as percentage similarity.

### ACE2 sequence alignments

ACE2 amino acid sequences were obtained from the UniProt database for human ACE2 (Q9BYF1), mouse ACE2 (Q8R0I0), rat ACE2 (Q5EGZ1), hamster ACE2 (A0A1U7QTA1), Bactrian camel ACE2 (A0A9W3H1F8), wild Bactrian camel ACE2 (A0A8B6YNL4), alpaca ACE2 (A0A6I9IHT2) and mink ACE2 (A0A8C7BTF2). Amino acid sequence alignment was generated using ClustalW (MEGA11 software). Percentage sequence similarity of animal ACE2 isoforms to human ACE2 was calculated using the bootstrap method (MEGA11 software; 1000 bootstrap replications; Poisson substitution model).

## Data Availability

The datasets used and/or analyzed during the current study available from the corresponding author on reasonable request.
